# Comparison of an automated and paper bladder diary in pediatric patients: mixed-methods, randomized crossover study

**DOI:** 10.3389/fped.2026.1959698

**Published:** 2026-10-08

**Authors:** Lola Bladt, An Bael, Nathalie Segers, Karolien De Moerloose, Katrien Klockaerts, Hendrik-Jan Florin, Lukas Van Campenhout, Stefan De Wachter, Alexandra Vermandel, Karen De Baets, Gunter De Win

**Affiliations:** 1 Product Development, Faculty of Design Sciences, University of Antwerp, Antwerp, Belgium; 2 Department of Pediatrics, Pediatric Nephrology, Queen Paola Children’s Hospital (ZAS), Antwerp, Belgium; 3 Department of Pediatrics, AZ Voorkempen, Emmaüs, Malle, Belgium; 4 Private Urology Practice, Aalst, Belgium; 5 Department of Urology, University Hospital Brussels, Brussels, Belgium; 6 Department of Urology, Imelda Hospital, Bonheiden, Belgium; 7 Department of Urology, University Hospital Antwerp, Antwerp, Belgium; 8 Antwerp Surgical Training, Anatomy and Research Centre (ASTARC), Faculty of Medicine and Health Sciences, University of Antwerp, Antwerp, Belgium; 9 Department of Rehabilitation Sciences and Physiotherapy, Faculty of Medicine and Health Sciences, University of Antwerp, Antwerp, Belgium

**Keywords:** automated bladder diary, child involvement, digital health, lower urinary tract symptoms, nocturnal enuresis, patient preference, pediatric urology, user experience

## Abstract

**Introduction:**

Bladder diaries are recommended for assessing pediatric lower urinary tract symptoms, but paper diaries require manual measurement, recording, and interpretation. Automated diaries may reduce this burden by combining automated volume measurement, digital registration, and guideline-based calculations. We compared an automated and a paper bladder diary regarding agreement, clinical utility, and user experience.

**Methods:**

In this mixed-methods, multicenter, randomized crossover study, children up to 13 years completed an automated diary (Minze Diary Pod) and paper diary in random order. Bland–Altman analysis assessed agreement; surveys and semi-structured interviews assessed clinical utility, user experience, and preferences.

**Results:**

Eighty participants were randomized; diary completion did not differ significantly between formats. Paired data were available for 49 children for frequency outcomes and 48 for volume outcomes (mean age 7.2 years, SD 2.0). The mean relative difference in average daytime voided volume was 3.2%, but the 95% limits of agreement (−48.5%–54.9%) exceeded the prespecified ±30% margin. Most frequency-based outcomes showed near-zero mean differences, with limits of agreement of approximately ±2–3 events per day. Classification differed between formats for 31.3% of participants for reduced bladder capacity and 23.3% for nocturnal polyuria, without statistically significant differences. Qualitative findings suggested that differences reflected day-to-day variation, self-monitoring effect, methodological differences between formats, including the variable measuring jugs used for paper diaries, and subjective symptom interpretation. Clinicians rated both formats clinically useful, but preferences were mixed across patients. Automated calculations were valued but not routinely consulted because of workflow constraints. Paper diaries remained appreciated for their familiarity and rapid visual overview, although guideline-based calculations were not consistently documented. The automated diary was preferred by 87.5% of parents and 72.9% of children, with significantly higher positive responses for motivation, pleasant use, child involvement, and perceived error-free use.

**Discussion:**

The automated diary showed strong usability and family preference, but individual-level measurement equivalence with the paper diary was not demonstrated. Without a true reference standard, disagreement did not establish which format was more accurate. Its standardized measurement and automated calculations may offer particular value for repeated monitoring, although workflow integration, clinician adoption of guideline-based calculations, and independent real-world evaluation remain necessary.

## 1 Introduction

Lower urinary tract symptoms (LUTS) and urinary incontinence are common in school-aged children and may occur during the day, at night, or both. Primary nocturnal enuresis (NE) affects approximately 10%–16% of 7-year-old children (1, 2), while daytime LUTS are reported in 4%–23% of school-aged children (3, 4). The International Children's Continence Society (ICCS) recommends a bladder diary for diagnostic assessment and treatment follow-up and considers it mandatory when history taking indicates daytime LUTS (5–8).

A bladder diary is a home-based assessment tool used to record voiding patterns in daily life over several days. In a conventional paper diary, children or caregivers measure each void using a measuring jug and manually record its time and volume, together with fluid intake, urgency, leakage episodes, and nighttime urine output, in a time-based paper table. It therefore combines quantitative volume measurements with manual registration and subjective symptom reporting to support diagnosis and treatment follow-up (5, 7).

According to ICCS guidelines, accurate interpretation of bladder diary findings, particularly in relation to the child's age-related expected bladder capacity (EBC), is important for guiding treatment (6). When daytime LUTS are present, these symptoms should generally be managed before NE is addressed. In children with monosymptomatic NE, EBC-based assessment of nocturnal polyuria and reduced bladder capacity can inform the choice of alarm therapy and/or desmopressin. Supporting this approach, a recent study reported that diary-guided treatment selection improved response by up to 46% (9).

Despite its clinical value, the conventional paper bladder diary remains burdensome for patients and clinicians. Patients and families must manually measure and record voided volumes using a jug, which can be cumbersome and may result in incomplete, unreliable, or low-quality data (10, 11). Clinicians, in turn, must perform several calculations to translate diary entries into clinically relevant parameters, particularly in children (6, 12, 13). This includes averaging voided volumes, calculating nocturnal urine production, including wet diaper weight when applicable, and interpreting these values relative to age-related EBC. Together, these steps make detailed and accurate paper diary interpretation time-consuming and potentially difficult to implement in routine clinical practice (14).

To overcome these limitations, several digital bladder diaries have been introduced. These diaries have shown promise as reliable alternatives to paper, with studies reporting improvements in usability and patient preference, and some suggesting improved compliance (14–23). They also enable automatic, guideline-based calculations and digital tracking of patient progress. In one study, digital diary interpretation time was almost halved compared with paper diaries, while accuracy in applying guideline definitions nearly doubled (19). However, digital diaries still depend on manual measurement of voided volumes using a jug, followed by digital entry. Consequently, they reduce the burden of manual recording but not the burden or potential inaccuracy of volume measurement, with reported measurement errors reaching or exceeding 10% of the actual voided volume (24–26).

More recent innovations in digital health have introduced automated bladder diaries, which may address this remaining limitation by automatically measuring voided volumes and transferring these data to a digital diary (27). Different technological approaches have been developed to achieve this automation. UScale (28) and the Uro-diary Checker (29) measure urine using a collection bag or cup combined with a separate measuring unit, whereas the Diary Pod (24, 30–34) integrates the collector and measuring sensor into a single device. Initial evaluations in adults have demonstrated promising accuracy and usability (28–34). However, pediatric evidence is lacking. Digital bladder diaries have been evaluated in only a limited number of pediatric studies (20, 23), and automated bladder diaries have, to our knowledge, not yet been assessed in children. This represents a relevant evidence gap, given the clinical value of bladder diaries in pediatric continence care and the specific compliance challenges faced by children and their families (23). Therefore, this study aimed to evaluate the Diary Pod, an automated bladder diary, in a pediatric population by comparing it with a conventional paper diary in terms of agreement, clinical utility, and patient and parent preference.

## 2 Materials and methods

### 2.1 Study design and settings

This mixed-methods, multicenter, randomized, two-period crossover study compared an automated bladder diary with a conventional paper bladder diary in terms of agreement, clinical utility, and user experience. The study was conducted across five pediatric nephrology or pediatric urology care settings in Belgium. Participants were randomized in a 1:1 ratio to one of two diary sequences: paper followed by automated diary, or automated followed by paper diary. Each diary was completed at home for at least two consecutive days. Diary periods were separated by an interval of at least two days, during which no changes in urological treatment were allowed.

The study protocol received ethical approval from the Ethics Committee of the University Hospital Antwerp and the University of Antwerp (BUN B3002023000056, 15 May 2023) and was registered at ClinicalTrials.gov (NCT06070675, https://clinicaltrials.gov/study/NCT06070675, 1 October 2023). No important protocol changes were made after study commencement. The reporting of this study was guided by CONSORT 2025 and the CONSORT 2010 extension for randomized crossover trials (see Consort Checklists in Supplementary Material).

### 2.2 Randomization and blinding

The allocation procedure was prepared by the first author, who was not part of the treating clinical team. No stratification was used, and allocation was balanced by preparing equal numbers of indistinguishable study packages for each diary sequence. At inclusion, clinicians blindly selected one package, thereby randomly assigning the participant to a diary sequence. Participants and clinicians were unaware of the assigned sequence before allocation.

To support adherence to the allocated sequence, the second diary format was stored in a locked document pouch, for which participants received the unlock code only after completing the first diary period and corresponding survey (Supplementary Figure S1). Blinding after allocation was not feasible because use and review of the two diary formats made them distinguishable.

### 2.3 Participants

Pediatric patients presenting to the participating clinics and identified by their treating clinician as requiring a bladder diary for diagnostic or therapeutic purposes were eligible. Children up to 13 years of age, both boys and girls, were included. Participants or their parents had to be able to use a smartphone or tablet. Exclusion criteria were inability to use the Diary Pod during voiding and inability to speak, read, or write Dutch.

Participants were recruited during routine outpatient visits. Eligible patients and parents received verbal and written study information. Written informed consent was obtained from parents or legal guardians; children aged 12 years and older also provided written informed consent, while younger children provided assent. Clinicians provided standardized verbal and written instructions for both diary formats and ensured correct setup of the automated diary.

### 2.4 Bladder diary procedures

#### 2.4.1 Paper bladder diary

The paper bladder diary (Figure 1) required manual recording of voids, urgency, fluid intake, and leakage episodes in a time-based table. In line with routine clinical practice, participants measured voided volumes using a self-provided measuring jug and recorded them manually. Paper diaries were reviewed and interpreted by clinicians during the follow-up visit.

**Figure 1:**
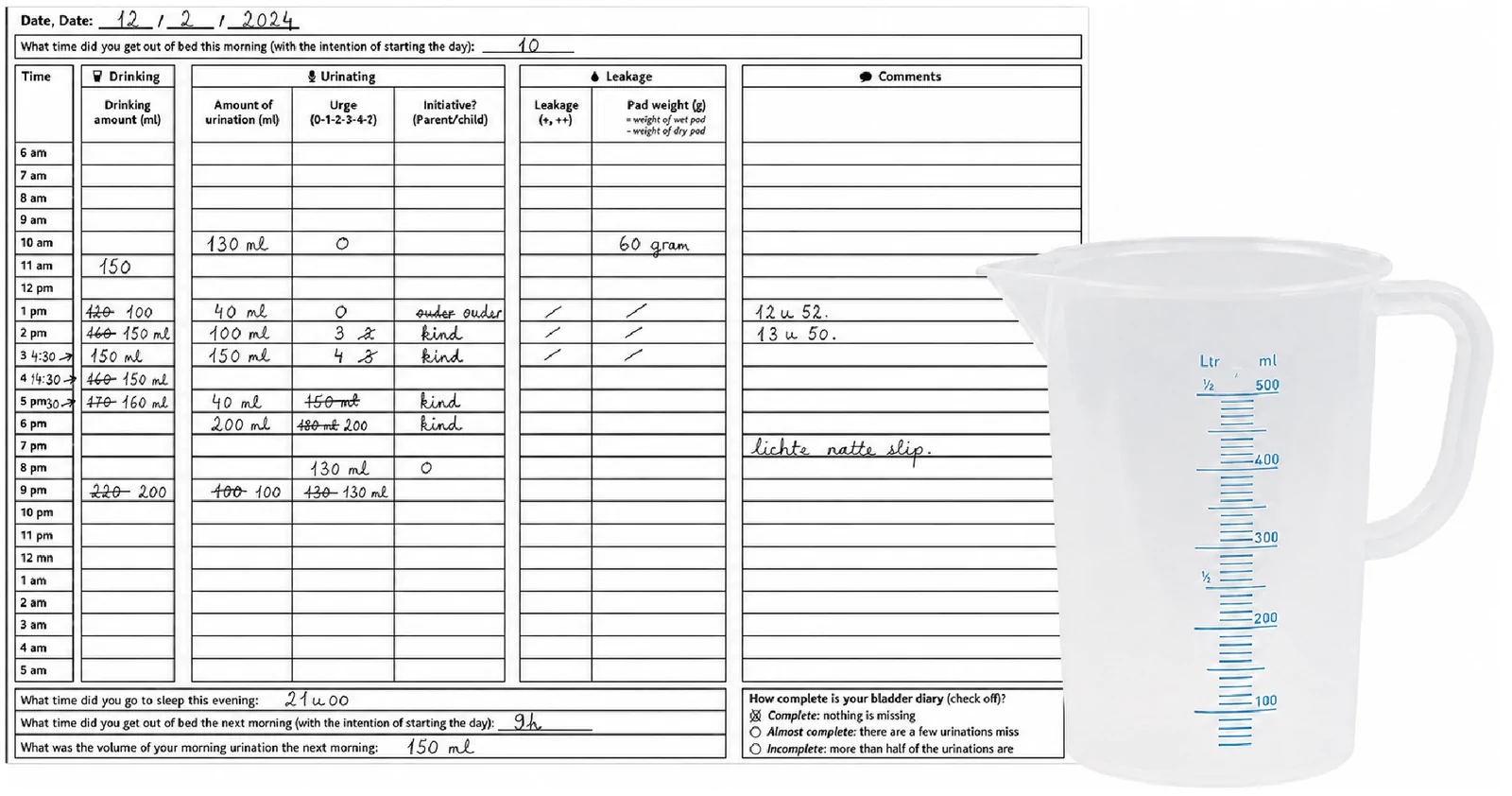
Paper bladder diary and measuring jug.

#### 2.4.2 Automated bladder diary

The automated bladder diary (Figure 2) consisted of the Diary Pod and the Minze Flow app (Minze Health, Antwerp, Belgium). The Diary Pod and Minze Flow app are CE-marked medical devices. The Diary Pod is a capacitance-based device that automatically measures voided volume. After each void, participants pressed a button on the device to register both the measured volume and the time of voiding. For this study, a handle was attached to facilitate use by children or parents during voiding. Data were transmitted via encrypted Bluetooth to the Minze Flow app, which prompted users to record additional information, including urgency, fluid intake, and leakage episodes. Automatic, guideline-based calculations and a timeline overview were made available for clinical review via the cloud-based portal. Automated diary data were pseudonymized and stored in the cloud, with personal identifiers and health data maintained separately. Data were encrypted during transmission and at rest, and access was restricted to authorized users in accordance with the General Data Protection Regulation.

**Figure 2:**
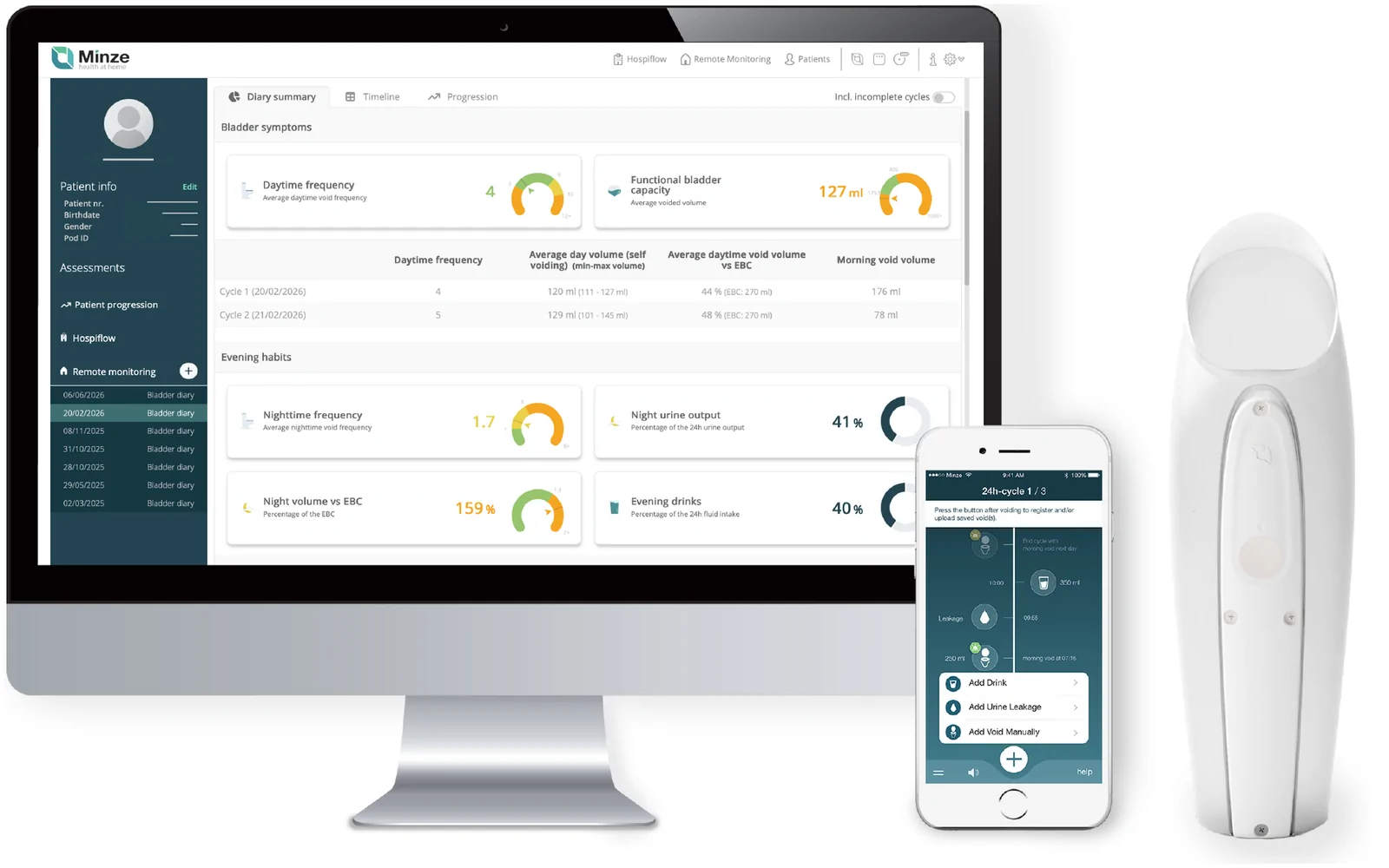
Automated bladder diary, including the Diary Pod, Minze Flow app, and clinician portal. Product images © Minze Health, reproduced with permission.

#### 2.4.3 Bladder diary parameters

Both diary formats collected the same variables: voiding times, voided volumes, urgency scores per void according to the International Consultation on Incontinence Questionnaire (ICIQ) bladder diary, fluid intake times and volumes, leakage episodes, and sleep and wake-up times. For analysis, automated diary data were exported directly from the cloud-based portal, while paper diary data were manually digitized into the same structured format. Diary parameters were calculated according to the definitions in Table 1 using a predefined analysis script. Parameters were calculated per diary cycle and averaged across cycles for each diary format.

**Table 1:** Definitions of bladder diary parameters and guideline-based classifications.

Parameter	Definition
Diary cycle	24h-period from the first morning void to the first morning void of the subsequent day
Average daytime voided volume	Mean voided volume during waking hours, excluding the first morning void after waking
Daytime frequency	Number of voids during waking hours, excluding the first morning void after waking
Nighttime frequency	Number of voids during sleeping hours
Urgency frequency	Number of voids with an urgency score >1, corresponding to greater than normal desire
Leakage frequency	Number of leakage episodes per 24h
Expected bladder capacity	Calculated as (age in years + 1) × 30 mL
Reduced bladder capacity	Maximum voided volume <65% of EBC
Nighttime urine production	Sum of voided volumes during the night, if any, the first morning voided volume, and wet diaper weight minus dry diaper weight, when applicable
Nocturnal polyuria	Nighttime urine production ≥130% of EBC

### 2.5 Clinician and patient/parent surveys

For each participant, clinicians completed a survey in which they rated both diary formats for quality on a 10-point Likert scale and clinical usefulness on a 5-point Likert scale, and indicated their preferred format, if any. Patients and parents completed an online survey after each diary format assessing usability using 5-point Likert-scale questions and diary format preference, if any. Both clinician and patient/parent surveys included open-ended questions for qualitative feedback.

The surveys were developed specifically for this study, drawing on the expertise of the supervising team, including a pediatric urologist, a urotherapist/physiotherapist, and a healthcare product design researcher. The content and wording were iteratively reviewed and internally tested by the research team until consensus was reached.

### 2.6 Semi-structured interviews

In addition, purposively selected subsamples of parents and clinicians participated in semi-structured interviews to further explore experiences with both diary formats. Interviews were audio-recorded with consent, transcribed verbatim, and pseudonymized before analysis.

### 2.7 Safety and harms

Given the non-invasive and non-therapeutic nature of the study, no formal adverse event monitoring or statistical analysis of harms was performed. Participants were instructed to report any adverse events to the treating clinician or study personnel during the study period.

### 2.8 Outcomes

The primary outcome was agreement between automated and paper bladder diaries for average daytime voided volume, expressed as the relative difference (%). The absolute volume difference (mL), daytime and nighttime voiding frequency, urgency frequency, and leakage frequency were assessed as secondary outcomes. Guideline-based classification agreement was assessed using predefined thresholds for reduced bladder capacity and nocturnal polyuria (Table 1). Additional secondary outcomes included clinician-reported diary quality, clinical usefulness, and diary format preference. Patient- and parent-reported outcomes included diary usability and diary format preference. Finally, qualitative data were used to contextualize findings on agreement, clinical utility, and user experience.

### 2.9 Statistical analysis

Agreement between automated and paper bladder diaries was assessed using Bland–Altman analysis, reporting mean differences and 95% limits of agreement. Agreement was additionally quantified using Lin's concordance correlation coefficient (CCC) for the continuous diary outcomes. Supplementary crossover analyses used linear mixed-effects models including diary method, period, and randomized sequence as fixed effects and participant as a random intercept. Paired binary outcomes were compared using McNemar's test, and paired ordinal outcomes using the Wilcoxon signed-rank test. All paired analyses included participants with available paired data for the outcome of interest using complete-case analysis; no imputation was performed. Independent categorical outcomes were compared using Fisher's exact test.

For analysis and visualization, 5-point Likert-scale usability responses were dichotomized into positive responses, defined as scores 4–5, versus all other responses. Negatively phrased items were reverse-coded so that higher scores consistently reflected a more positive user experience. All quantitative data analyses were conducted using R version 4.5.2 (R Foundation for Statistical Computing, Vienna, Austria; RRID:SCR_001905) within RStudio (Posit Software, PBC, Boston, MA, USA; RRID:SCR_000432).

Qualitative data from open-ended survey responses and semi-structured interviews were analyzed thematically using a deductive coding framework informed by the quantitative findings, while allowing additional themes to emerge inductively from the data.

Data extraction and both the quantitative and qualitative analyses were performed by the first author. Blinding during data extraction and analysis was not feasible because the diary format was evident from the source material.

### 2.10 Sample size

The sample size was determined to ensure adequate precision for estimating agreement between diary methods using Bland–Altman analysis. Based on average daytime voided volume data from three prior diary comparison studies (18, 19, 22), the expected mean relative difference between methods was 0.6%, with a standard deviation of 10.6%. Using a prespecified reference agreement margin of ±30%, at least 47 paired observations were required. Allowing for 40% dropout or incomplete diary data based on previous studies (20, 22, 23), the target sample size was set at 80 participants. No interim analyses or stopping guidelines were planned.

## 3 Results

### 3.1 Participant flow and baseline characteristics

Participants were recruited between October 2023 and June 2025, and final follow-up was completed in September 2025. Eighty eligible patients agreed to participate, provided informed consent, and were randomized: 40 (50%) to the paper-first sequence and 40 (50%) to the automated-first sequence. Paired diary data were available for 49 participants for frequency-based parameters and for 48 participants for voided volume-related parameters, as one paper diary had missing voided volumes (Figure 3).

**Figure 3:**
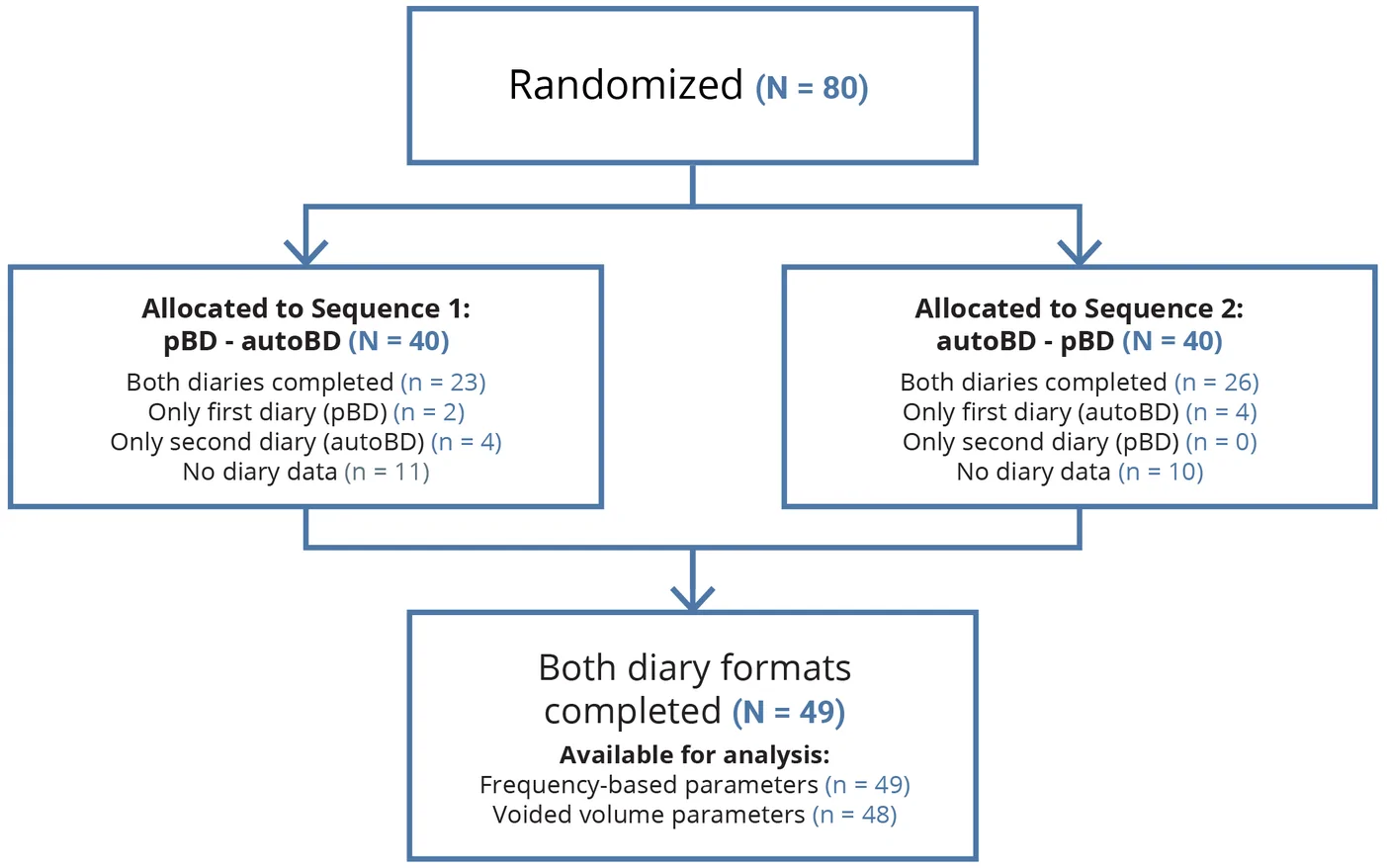
Flow diagram of participant allocation, diary completion, and paired diary data availability in the randomized crossover study. pBD, paper bladder diary; autoBD, automated bladder diary.

Noncompletion, defined as no data or fewer than two days of recordings in one or both diary formats, resulted in 38.8% (31/80) of randomized participants without analyzable paired diary data, consistent with the anticipated dropout rate. Completers and non-completers were descriptively similar in age (mean 7.2 ± 2.0 vs. 7.5 ± 1.9 years) and sex distribution (63.3% vs. 58.1% boys). Among completers, 53.1% (26/49) had been allocated to the automated-diary-first sequence, compared with 45.2% (14/31) of non-completers. Reasons for noncompletion were unavailable for most participants, generally because families did not return for follow-up or provide further information. Documented reasons included a technical issue with the automated diary, absence of a suitable smartphone, a lost paper diary, and difficulty completing the paper diary.

Completion was numerically higher for the automated diary than for the paper diary (71.3% [57/80] vs. 63.8% [51/80]; McNemar *p* = 0.11) and among participants who started with the automated diary than among those who started with paper (65.0% [26/40] vs. 57.5% [23/40]; Fisher's exact *p* = 0.65). These findings did not indicate a clear association between completion and diary format or randomized sequence.

Clinician ratings were completed for 49 participants with both diary formats available for review. Patient/parent surveys were completed by 58 and 48 participants after the first and second diary periods, respectively. Paired usability analyses included 47 participants who provided responses for both diary formats, while preference data were available for 48 participants. Semi-structured interviews were conducted with 13 parents and 2 clinicians. No adverse events were reported.

Participant characteristics are presented in Table 2. Among participants with paired diary data, the mean age was 7.2 years (SD 2.0; range 5–13). Nearly three quarters completed the diaries in the context of a first consultation (36/49, 73.5%), and most had no prior experience completing a bladder diary (38/49, 77.6%). Participants presented with a range of urinary complaints, most commonly NE (41/49, 83.7%) and daytime urinary incontinence (24/49, 49.0%). Approximately half of the children owned a smartphone or tablet, and a similar proportion reported daily use of these devices.

**Table 2:** Baseline characteristics of all randomized participants and participants with paired diary data, by randomized sequence.

Characteristics		Sequence 1 pBD - autoBD	Sequence 2 autoBD - pBD	Total
All randomized participants		*n* = 40	*n* = 40	*N* = 80
Age in years, mean ± SD		7.4 *±* 2.3	7.1 *±* 1.7	7.3 *±* 2.0
Sex, *N* (%)	Boy	27 (67.5)	22 (55.0)	49 (61.3)
	Girl	13 (32.5)	18 (45.0)	31 (38.8)
Participants with paired diary data		*n* = 23	*n* = 26	*n* = 49
Age in years, mean ± SD		7.4 *±* 2.2	7.1 *±* 1.7	7.2 *±* 2.0
Sex, *N* (%)	Boy	16 (69.6)	15 (57.7)	31 (63.3)
	Girl	7 (30.4)	11 (42.3)	18 (36.7)
First consultation, *N* (%)		17 (73.9)	19 (73.1)	36 (73.5)
Not familiar with filling out a bladder diary, *N* (%)		16 (69.6)	22 (84.6)	38 (77.6)
Presenting complaints, *N* (%)	NE	17 (73.9)	24 (92.3)	41 (83.7)
	DUI	12 (52.2)	12 (46.2)	24 (49.0)
	Frequency	9 (39.1)	7 (26.9)	16 (32.7)
	Urgency	9 (39.1)	11 (42.3)	20 (40.8)
	Dysuria	2 (8.7)	1 (3.8)	3 (6.1)
	UTI	0 (0)	2 (7.7)	2 (4.1)
	Constipation	2 (8.7)	3 (11.5)	5 (10.2)
	FI	3 (13.0)	1 (3.8)	4 (8.2)
	Other	1 (4.3)	0 (0)	1 (2.0)
Child has their own smartphone or tablet, *N* (%)		9 (39.1)	15 (57.7)	24 (49.0)
Child uses a smartphone or tablet each day, *N* (%)		11 (47.8)	16 (61.5)	27 (55.1)

DUI, daytime urinary incontinence; FI, fecal incontinence; NE, nocturnal enuresis; SD, standard deviation; UTI, urinary tract infection.

### 3.2 Agreement between automated and paper diaries

For the primary outcome, the mean relative difference (%) in average daytime voided volume between automated and paper diaries was 3.2% (95% CI −4.4%–10.9%; *N* = 48). The lower and upper limits of agreement were −48.5% (95% CI −61.7%–−35.3%) and +54.9% (95% CI 41.8%–68.1%), respectively (Figure 4). The predefined ±30% agreement criterion was therefore not met, indicating substantial individual-level variability.

**Figure 4:**
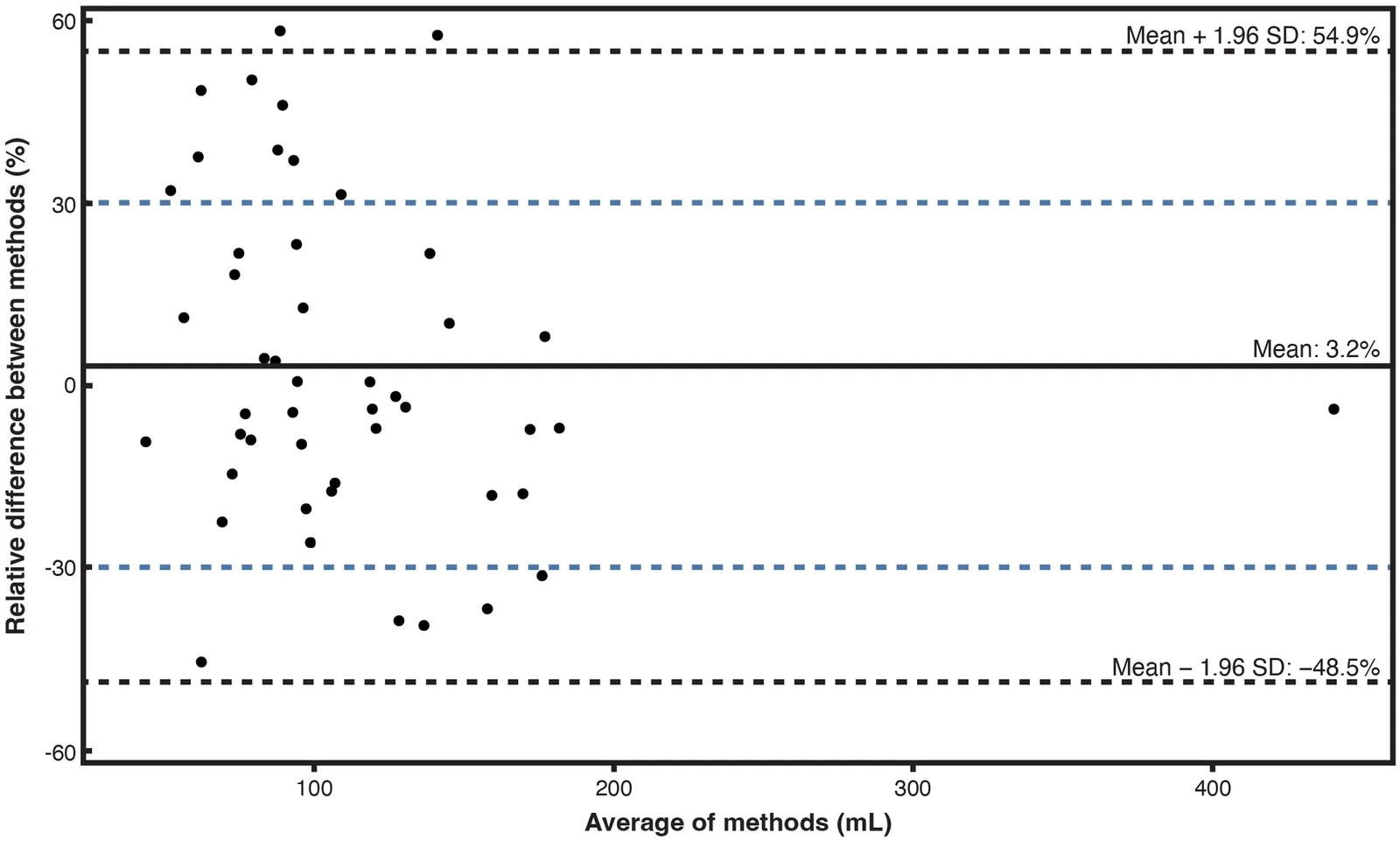
Bland-Altman plot of the relative difference (%) in average daytime voided volume between automated and paper bladder diaries (*N* = 48), calculated as (automated−paper)/paper × 100%. The solid black line represents the mean relative difference, dashed black lines indicate the 95% limits of agreement, and dashed blue lines indicate the predefined ±30% agreement margin.

For secondary diary outcomes, Bland–Altman results and Lin's CCC are presented in Table 3. When average daytime voided volume was assessed on the absolute scale (mL), the mean between-method difference was −2.9 mL (95% CI −11.1–5.3 mL), with limits of agreement ranging from −58.2 to 52.4 mL. Frequency-based outcomes showed mean differences close to zero, while the limits of agreement indicated individual differences of approximately ±2–3 events per day for most outcomes. Urgency frequency showed the widest limits of agreement (−4.9–2.7 events per day) and a mean difference corresponding to approximately one fewer void with reported urgency per day in the automated diary. The variation in agreement across outcomes was also reflected in Lin's CCC estimates. Concordance was highest for average daytime voided volume (0.896), lower for daytime and leakage frequencies (0.616 and 0.548, respectively), and lowest for nighttime and urgency frequencies (0.234 and 0.295, respectively).

**Table 3:** Agreement analyses for bladder diary outcomes between automated and paper bladder diaries.

Parameter	*N*	Mean difference (95% CI)	SD	Lower LoA (95% CI)	Upper LoA (95% CI)	Lin's CCC (95% CI)
Primary endpoint (relative difference)						
Relative average daytime voided volume	48	3.2% (−4.4 to 10.9%)	26.4%	−48.5% (−61.7 to −35.3%)	54.9% (41.8 to 68.1%)	–
Absolute difference						
Average daytime voided volume	48	−2.9 mL (−11.1 to 5.3 mL)	28.2 mL	−58.2 mL (−72.2 to −44.1 mL)	52.4 mL (38.3 to 66.5 mL)	0.896 (0.825 to 0.939)
Frequency-based (counts)						
Daytime frequency	49	0.03 (−0.4 to 0.4)	1.4	−2.8 (−3.5 to −2.1)	2.9 (2.1 to 3.6)	0.616 (0.409 to 0.763)
Nighttime frequency	49	0.1 (−0.1 to 0.3)	0.7	−1.2 (−1.5 to −0.9)	1.5 (1.1 to 1.8)	0.234 (−0.028 to 0.466)
Urgency frequency	49	−1.1 (−1.7 to −0.6)	1.9	−4.9 (−5.9 to −4.0)	2.7 (1.7 to 3.7)	0.295 (0.074 to 0.488)
Leakage frequency	49	−0.1 (−0.4 to 0.2)	1.0	−2.1 (−2.6 to −1.6)	1.9 (1.4 to 2.4)	0.548 (0.330 to 0.711)

Differences were calculated as automated minus paper. Relative difference was calculated as (automated−paper)/paper × 100%. Limits of agreement were calculated as the mean difference ± 1.96 SD.

CCC, concordance correlation coefficient; CI, confidence interval; LoA, limits of agreement; SD, standard deviation.

The supplementary crossover analyses similarly identified a method effect only for urgency frequency, which was lower in the automated diary (−1.1 events per day, 95% CI −1.7–−0.6; *p* = 0.0002). No period effects (*p* = 0.075–0.563) or sequence effects (*p* = 0.303–0.776) were observed for any diary outcome (Supplementary Table S1).

Classification agreement was 68.8% (33/48; 13 positive and 20 negative by both formats) for reduced bladder capacity and 76.7% (33/43; 4 positive and 29 negative by both formats) for nocturnal polyuria. Discordant classifications occurred in 15/48 participants (31.3%; 9 positive by the automated diary only and 6 by the paper diary only) for reduced bladder capacity and 10/43 participants (23.3%; 4 positive by the automated diary only and 6 by the paper diary only) for nocturnal polyuria. Most discordances for reduced bladder capacity occurred near the predefined cutoff, with 13/15 (86.7%) discordant cases within ±10% of the threshold in at least one diary format. For nocturnal polyuria, 5/10 (50.0%) discordant cases were within ±10% of the threshold. McNemar's tests showed no evidence of systematic classification differences between formats for either reduced bladder capacity (*χ*^2^ = 0.27, *p* = 0.61) or nocturnal polyuria (*χ*^2^ = 0.10, *p* = 0.75).

Qualitative findings suggested that, despite differences in individual diary values, clinicians often derived similar overall clinical interpretations from the two formats (Table 4). Reported explanations for variability included day-to-day contextual factors, a potential self-monitoring effect after the first diary, methodological differences between formats, and subjective symptom interpretation. Methodological differences mainly reflected variability in measuring jugs used for paper diaries (Figure 5), while urgency ratings were especially prone to subjective scoring, with some parents using their own understanding of the urgency scale. This was particularly reported for paper diaries, where the score definitions were provided only in the written instructions, whereas the app displayed them with each entry. These qualitative findings provide context for the difference in urgency frequency observed between formats.

**Table 4:** Qualitative themes related to agreement between diary formats.

Themes	Illustrative quotes
Comparable clinical interpretations	“I don't think there were major differences. Both the manual and computer-based results actually led to the same conclusions [according to the doctor].” (P47; mother, 34 years; boy, 7 years)
	“The Diary Pod measures more accurately than the measuring jug, but for the majority of patients I found both diaries comparable and both clinically useful. Whether it is suddenly 256 instead of 250 mL does not really change the interpretation.” (Clinician 5)
Day-to-day contextual variability	“It was a very warm and active day during the paper diary, so he drank much more than usual. He had also gone to a birthday party and drank lemonade and cola, which he normally would not be allowed to drink at home.” (P14; mother, 37 years; boy, 7 years)
	“I notice that emotional factors really affect my child. When he is stressed or overwhelmed, the symptoms occur much more quickly, for example during the first nights after he returns from his father's home.” (P27; mother, 39 years; boy, 8 years)
Potential self-monitoring effect	“Because we had already been paying more conscious attention to it, his urination and fluid intake behavior may have changed.” (P21; mother, 37 years; boy, 7 years)
	“There were far fewer accidents during the second bladder diary, although we could not really explain why.” (P72; mother, 40 years; boy, 5 years)
Methodological differences	“With the larger measuring jug [used for the paper diary], his voided volumes suddenly seemed much bigger. But when we poured 200 mL from one jug into a smaller jug, it turned out to be much less. With the Diary Pod, measurements are always registered in the same way. I think this makes comparisons much more reliable.” (P14; mother, 37 years; boy, 7 years)
	“Not all measuring jugs have equally accurate scale markings for small voided volumes, for example below 100 mL.” (P49; father, 49 years; boy, 5 years)
	“With the Diary Pod, the timing felt much more accurate. If you pressed the button at 11:14, it was recorded exactly at 11:14. In the paper diary, we just wrote it within the hourly block.” (P80; mother, 43 years; girl, 7 years)
Subjective symptom interpretation	“[When completing the paper diary,] I asked whether he needed to urinate urgently and to rate it on a scale from 1 to 10, but he always answered ‘10’. It felt very subjective and open to interpretation.” (P47; mother, 34 years; boy, 7 years)
	“Parents interpret urgency scores very differently, and you really have to ask additional questions to understand what they actually mean. We are very aware ourselves of how subjective this interpretation is.” (Clinician 2)

**Figure 5:**
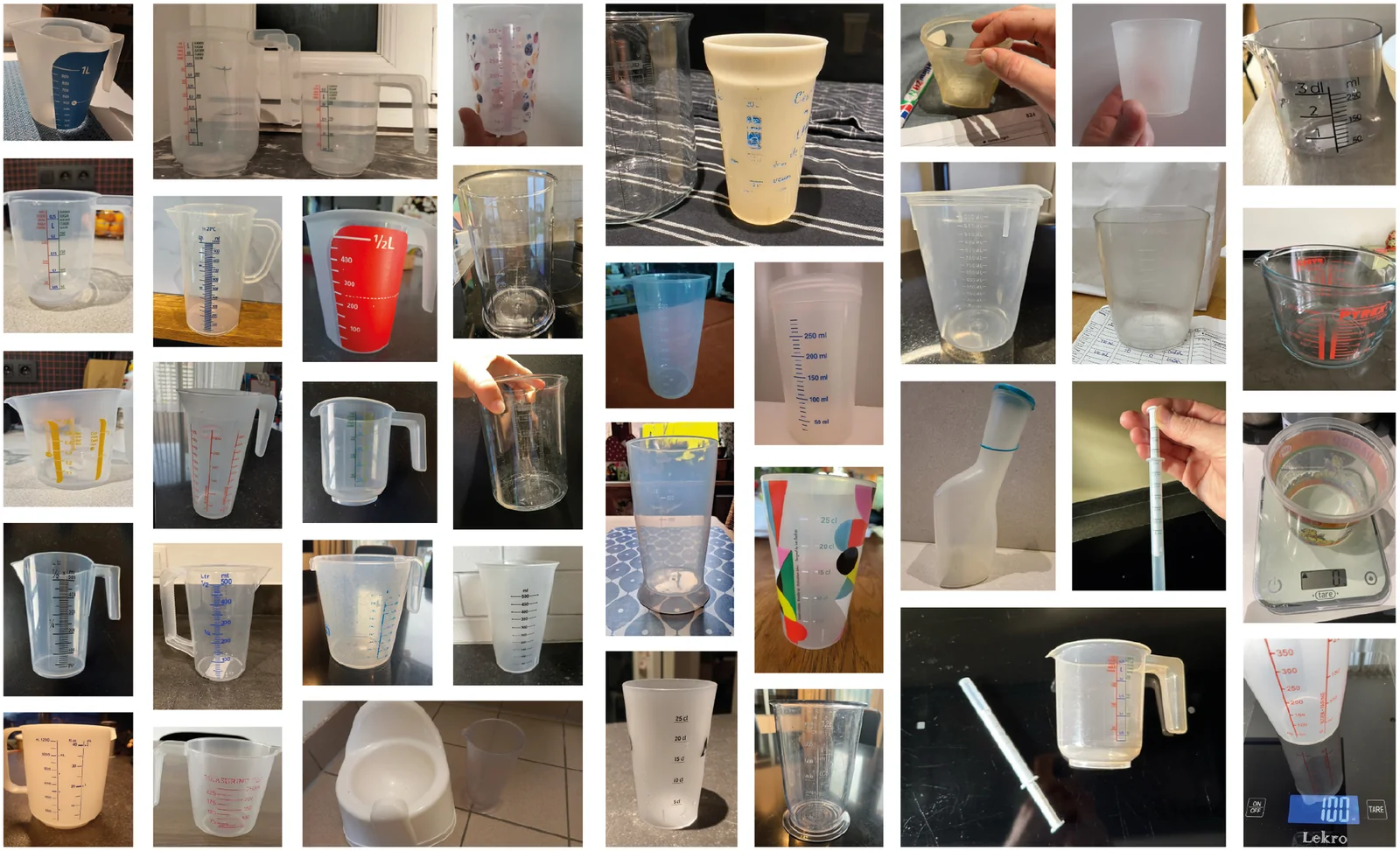
Variability in measuring jugs used during paper bladder diary completion. Photographs submitted by participants through the study survey and reproduced with consent.

### 3.3 Clinical utility

Clinicians rated both diary formats highly. The automated diary received slightly higher quality ratings than the paper diary, although this difference was not statistically significant (median 9, interquartile range [IQR] 8–9, vs. median 8, IQR 7–9; *p* = 0.11). Clinical usefulness ratings were similarly high for both formats (median 5, IQR 4–5 for both; *p* = 0.44). When asked to indicate an overall preference per patient, clinician preferences were mixed: the automated diary was preferred in 19/49 cases (39%), the paper diary in 12/49 cases (24%), and no preference was reported in 18/49 cases (37%). Qualitative findings further showed that preferences were influenced by data completeness, perceived reliability, readability, and whether diary completion coincided with atypical or hectic days, rather than by diary format alone (Table 5).

**Table 5:** Qualitative themes related to clinician preferences and clinical utility.

Themes	Illustrative quotes
Diary preference influenced by data quality	“For this patient, it did not really matter. Both diaries were very carefully and completely filled out. I think the added value of the automated diary mainly lies with parents who are less precise, more nonchalant, or more forgetful.” (Clinician 1)
	“The automated diary was recorded much more accurately than the paper diary. I am not sure whether this was due to the format itself or because the paper diary happened to be completed during very hectic days.” (Clinician 1)
	“The automated bladder diary is much easier to read.” (Clinician 3)
Automated calculations and data accessibility	“Too much manual counting with the paper diary and it was difficult to quantify progress. The automated diary provided a clear overview and the calculations were very useful.” (Clinician 2)
	“Are the calculations actually available on the paper diary? Half the time you cannot find them. That is especially true for residents, who do not yet know which parameters are important and why.” (Clinician 4)
	“The paper diaries need to be scanned. That can take up to two months, and they can also get lost. Digital data remain directly accessible.” (Clinician 4)
Workflow challenges and paper familiarity	“When reviewing the automated diary on the computer screen, I often needed to click a lot. We have been used to reviewing paper diaries for so long that we can quickly go through them and immediately pick out what we need.” (Clinician 4)
	“It is a huge amount of paper and I know it is outdated, but I am so used to it. Searching everything on a computer feels more time-consuming.” (Clinician 5)

Although preferences depended on the individual diary and patient context, clinicians reported several perceived advantages of the automated format. It was perceived as more readable and particularly useful for parents who were less precise or consistent in recording diary data. Digital accessibility was considered an important advantage, making diaries easier to retrieve, whereas paper diaries could get lost or be unavailable at follow-up. Moreover, automated calculations could reduce manual counting and support more consistent, guideline-based interpretation, as some parameters might otherwise be interpreted differently or overlooked depending on the clinician or resident reviewing the diary. The availability of consistently calculated parameters across diaries, together with automated comparisons of progress over time, was also identified as a potential benefit for longitudinal follow-up and the assessment of treatment effectiveness.

However, the findings also revealed a gap between the availability and clinical use of guideline-based calculations. Although these calculations were generated automatically by the automated diary, clinicians did not routinely consult them because of additional computer interactions, established workflows, and time pressure. In contrast, paper diaries remained appreciated for their familiarity and rapid visual overview during face-to-face consultations. Yet guideline-based calculations and classifications were not consistently documented for paper diaries in the clinician surveys, suggesting that this rapid, pattern-based visual review may not always have been accompanied by systematic calculation and guideline-based interpretation.

### 3.4 User experience

Parent-reported experience outcomes for the 47 participants with paired usability responses are summarized in Figure 6 and Supplementary Table S2. Positive response percentages were higher for the automated diary across all outcomes. Significantly more parents reported positive responses for motivation (87.2% vs. 72.3%; *p* = 0.039), pleasant use (72.3% vs. 25.5%; *p* < 0.001), child involvement (100.0% vs. 76.6%; *p* < 0.001), and perceived error-free use (53.2% vs. 27.7%; *p* = 0.012).

**Figure 6:**
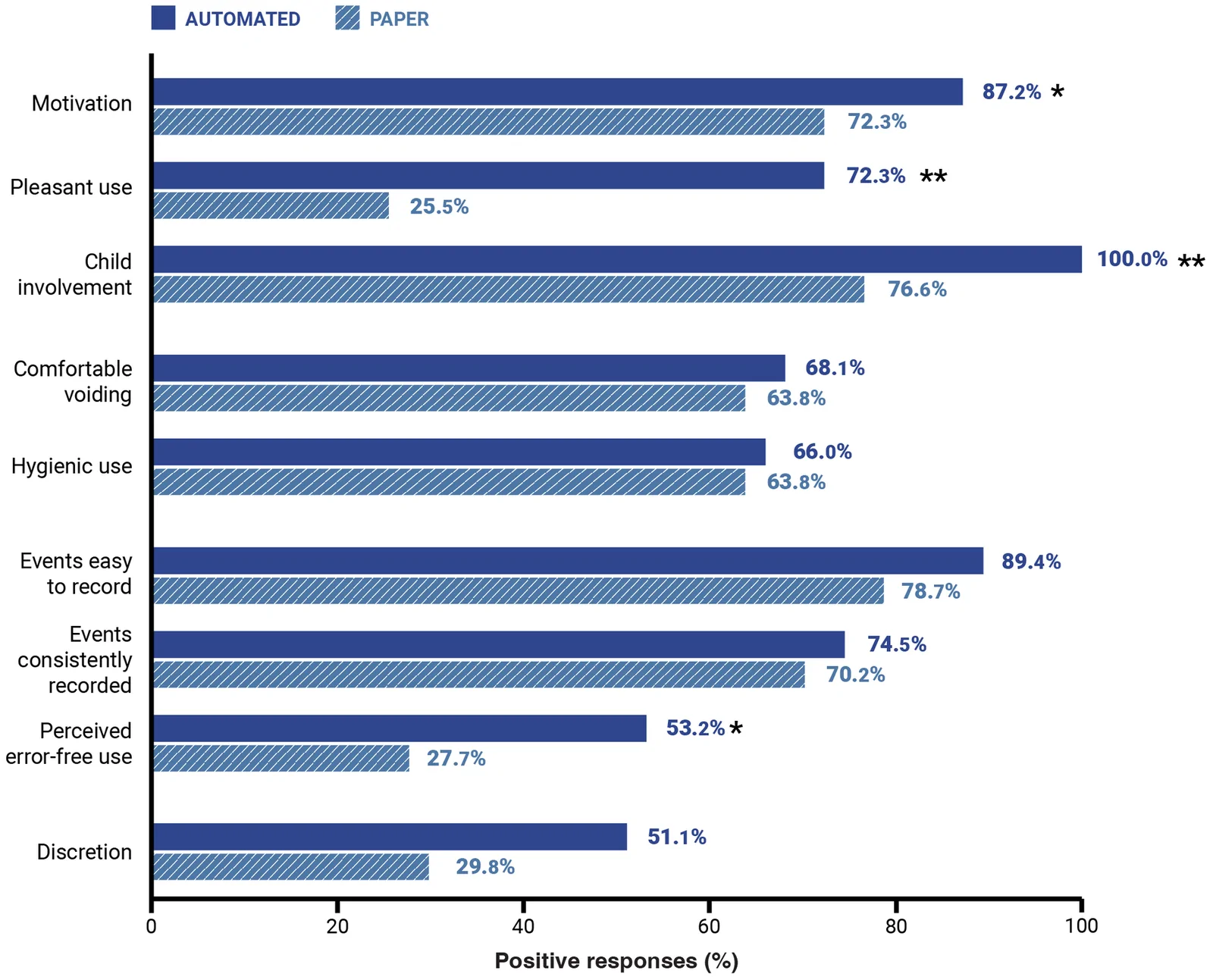
Parent-reported experience with the automated and paper bladder diaries among the 47 participants with paired usability outcomes. Bars show the percentage of positive responses for each survey item. Statistical comparisons were performed using McNemar's tests. Exact *p*-values are reported in Supplementary Table S2. *p* < 0.05 (*), *p* < 0.01 (**).

Qualitative findings contextualized these user experience outcomes (Table 6). Although motivation was high for both formats and was mainly linked to the desire to improve the child's urinary symptoms and support participation in social activities, the diary format also played a role. Some parents described the extensive paper tables as cumbersome or overwhelming, which reduced their motivation to complete the diary. In contrast, the automated format was perceived as more engaging and interactive: children could use the Diary Pod, press the button, interact with the app, and view the measured volume, which often made them curious. In contrast, the paper diary was more parent-led because measuring and recording volumes with a measuring jug often required parental assistance. Some participants also perceived the Diary Pod as more hygienic and less prone to spilling than the measuring jug, although its length made seated voiding difficult for some children.

**Table 6:** Qualitative themes related to user experience and preference.

Themes	Illustrative quotes
Motivation to complete the diary	“She wanted to get rid of the bedwetting problem so she could go for sleepovers without having to make excuses. For her, it was like: we need to complete the diaries, otherwise the doctors cannot help me.” (P80; mother, 43 years; girl, 7 years)
	“When I saw that stack of paper [the paper diary], really like one big table to complete, I honestly thought: ‘I do not want to do this at all.’ My motivation completely sank. It simply felt overwhelming.” (P27; mother, 39 years; boy, 8 years)
Engagement and child involvement	“He was very engaged and always wanted to do it himself. He enjoyed pressing the button, checking the volumes, and interacting with the app. Looking back, he probably could have perfectly managed everything independently.” (P27; mother, 39 years; boy, 8 years)
	“He could press the button and it connected automatically, which made him much more involved than when I had to read and write everything down myself. Reading the lines on the measuring cup was also more difficult for him.” (P77; mother, 47 years; boy, 7 years)
	“The paper diary lacked ‘playfulness’; it was a very strict document, making it harder to involve the child.” (P41; mother, 43 years; boy, 7 years)
Hygiene and urine collection	“With the measuring jug [used for the paper diary] she had to spread her legs wider because it is round; it is not the right shape. Personally, I was worried about spilling it on my hands. So I found it less hygienic and less comfortable [than the Diary Pod].” (P54; mother, 42 years; girl, 6 years)
	“She usually used the Diary Pod while standing. She was supposed to sit, but the Diary Pod is simply too long. If it could hang in the toilet, we would probably like it more.” (P17; mother, 40 years; girl, 9 years)
Data registration and errors	“With the paper diary, I think two or three times I wrote something in the wrong column, like drinks under voiding or vice versa. In the app, that kind of mistake was much less likely because it felt more intuitive.” (P72; mother, 40 years; boy, 5 years)
	“The information was more bundled in the app because it asked follow-up questions that you might not think of recording on paper. You had to answer them immediately, so you were less likely to forget.” (P47; mother, 34 years; boy, 7 years)
	“The fact that everything was immediately available in the app with the correct timestamp made it very user-friendly. Basically, you only had to enter the drinks. However, remembering to register drinks still required some extra attention.” (P55; mother, 37 years; boy, 7 years)
	“The app was installed on my phone but not on my husband's. Once I was at a restaurant and our child was not with me, so we ended up entering the information manually afterwards.” (P79; mother, 41 years; boy, 6 years)
Portability and discretion	“With the paper diary, you are always carrying around a sheet of paper. With a smartphone, everyone is on their phone anyway, so it attracts much less attention when entering information.” (P14; mother, 37 years; boy, 7 years)
	“I always kept the Diary Pod in its pouch and carried it in my bicycle bag wherever we went.” (P72; mother, 40 years; boy, 5 years)
Willingness to pay and perceived value	“For a bladder diary over two or four days, I would not buy it, because I can also commit to filling in the paper version. But if it were part of a training program or linked to physiotherapy, no problem.” (P47; mother, 34 years; boy, 7 years)
	“It would be good if you could see her bladder capacity increasing and follow the results in graphs. Something child-friendly could be added as well, perhaps a character that presents the results from a child's perspective. Then you are not only paying for the device, but also for the app.” (P54; mother, 42 years; girl, 6 years)

Automated registration of voided volumes and timestamps was perceived as reducing burden and errors. By prompting follow-up questions at the time of registration, the automated diary encouraged real-time data entry and more complete recording. Smartphone-based registration also made the diary portable and discreet, especially outside the home. In contrast, the paper diary was easier to forget, less portable, and more prone to delayed, incomplete, or incorrect registration. However, app-based registration also introduced challenges related to dependence on a parent's phone, involvement of multiple caregivers, and remembering manual fluid intake registration despite automated urine registration.

Preferences reflected the more favorable user experience with the automated diary. Most parents preferred the automated diary (42/48, 87.5%), as did most children (35/48, 72.9%). Although affordability remained a concern, willingness to pay appeared higher when the diary was perceived as part of a longer-term therapy or training trajectory rather than as a short-term diary assessment.

## 4 Discussion

### 4.1 Principal findings

Average differences between the automated and paper diaries were generally small at the group level, but the prespecified ±30% agreement margin at the individual participant level was not met. Qualitative findings suggested that this individual variability may partly reflect the sensitivity of bladder diary outcomes to real-life day-to-day context and methodological differences between formats, including the substantial variation in self-provided measuring jugs used for paper diaries.

Clinicians nevertheless generally derived comparable overall clinical interpretations from both formats and rated both clinically useful, although their preferences were mixed across patients. The findings also showed a gap between the availability and clinical use of guideline-based calculations. Automatically generated outputs were not routinely consulted, while corresponding calculations and classifications were not consistently documented for paper diaries.

Families showed a clear preference for the automated diary, with most parents and children preferring this format. User experience was more favorable across all evaluated outcomes, and significant differences were observed for motivation, pleasant use, child involvement, and perceived error-free use. However, overall diary completion did not differ significantly between formats.

### 4.2 Measurement agreement and clinical interpretation

Previous studies comparing digital, automated, and paper bladder diaries have reported mixed findings regarding agreement, completeness, and compliance (17–23, 29, 33–35). Our findings similarly revealed a nuanced picture. Although average differences between formats were small at the group level, individual-level agreement varied considerably across participants: the limits of agreement for average daytime voided volume exceeded the prespecified ±30% margin, differences in frequency-based outcomes extended to approximately ±2–3 events per day, and classifications differed between formats for approximately one-third of participants for reduced bladder capacity and one-quarter for nocturnal polyuria. The findings therefore indicate that the two formats do not always produce equivalent results at the individual participant level. Some of these differences may be clinically meaningful for individual children. Nevertheless, the observed numerical and classification differences did not necessarily affect clinicians’ overall assessments, possibly because diary findings were considered alongside other assessments and because guideline-based calculations were not consistently consulted or documented. Moreover, among the discordant classifications, 13 of 15 for reduced bladder capacity and half for nocturnal polyuria involved at least one value close to the respective cutoff.

However, this comparison does not validate one method against the other. The paper diary served as the conventional comparator but not as a reference standard. Its recognized burden and susceptibility to incomplete, unreliable, or low-quality data should therefore be considered when interpreting disagreement between methods (10, 11). In the absence of a true reference standard, which is difficult to obtain in home-based bladder diary studies, disagreement between diary formats cannot be definitively attributed to one format being more accurate than the other. Our qualitative findings suggest that such differences may reflect a combination of day-to-day contextual variation, a potential self-monitoring effect, differences in methodology and participant engagement between formats, and subjective symptom interpretation. This aligns with previous bladder diary research showing substantial day-to-day variability in diary parameters (36, 37) and suggesting that diary completion itself may influence behavior (38). This self-monitoring effect is also reflected in the use of bladder diaries as part of urotherapy for children with daytime symptoms (39). Self-monitoring may thus have affected individual families, even though no consistent period or sequence effects emerged at the group level. In a two-period, two-sequence crossover design, however, any resulting carryover is difficult to distinguish from other order-related effects.

Although the present study cannot establish which format was more accurate under real-life conditions, the underlying measurement processes differ substantially. The Diary Pod has previously been evaluated for automated volume measurement in controlled bench testing against a calibrated laboratory scale (30). In line with routine practice at the participating centers, participants used self-provided measuring jugs for the paper diary. Paper diaries therefore depend on the accuracy and scale resolution of the measuring jug, manual reading and estimation, and subsequent written registration. The substantial variation observed in the measuring jugs used by participants represents an important real-world finding and highlights a potential source of measurement variability that clinicians should consider when interpreting paper diaries. A previous study similarly reported discrepancies in manually measured voided volumes (24). Furthermore, the automated diary registered the measured volume and time immediately after participants pressed the button following each void, thereby reducing reliance on later recall and transcription. This standardized measurement and registration process may reduce sources of measurement variability associated with paper diaries, although superior accuracy under real-life conditions cannot be established from the present study.

### 4.3 Clinical use of automated calculations

Automated calculations may improve the consistency and efficiency of bladder diary interpretation. Guideline-based interpretation of bladder diaries requires several calculation steps, including averaging voided volumes, calculating nocturnal urine production, and interpreting results relative to age-related EBC (6). Performing these steps manually can be challenging under clinical time pressure. Previous research showed that automated calculations almost halved interpretation time and nearly doubled the correct application of guideline definitions compared with manual interpretation of paper diaries (19).

However, our findings suggest that guideline-based calculations remain underused in routine clinical practice. For the automated diary, the calculations were generated automatically but not routinely consulted because of established habits, additional computer interactions, time pressure, and workflow constraints. Paper diaries, by contrast, continued to be valued for their familiarity and rapid visual overview, although the calculations had to be performed manually and the resulting parameters and classifications were not consistently documented. Together, these findings indicate a gap in both workflow integration and clinical review practices. This gap may also help explain why numerical differences between formats did not necessarily alter clinicians’ overall interpretations. Automation removes the burden of manual calculation, but its clinical value cannot be fully realized unless clinicians systematically use the resulting outputs.

Realizing the potential value of automated calculations will require improved integration into routine care. Calculated parameters should be immediately visible, rapidly interpretable, and accessible within clinicians’ existing workflows. Printable summaries, electronic health record integration, and decision-support features may support this process. Further human-factors and behavioral research involving clinicians could help determine how these outputs should be presented and integrated to encourage their systematic use. Making these guideline-recommended calculations more accessible could complement clinical expertise and facilitate their use without adding to the consultation burden, thereby supporting more consistent guideline-based management across patients.

### 4.4 Patient and parent preference

Patient preference appears more consistent across previous studies than agreement or compliance, with several studies reporting preference rates of 80% or higher for digital or automated formats (17, 19, 33, 35). Our findings support this pattern: most parents and children preferred the automated diary, and their experience was more favorable across all evaluated usability outcomes.

Child involvement was significantly higher with the automated diary. This contrasts with previous pediatric studies reporting similar child participation between digital and paper formats (20, 23). Pressing the button, interacting with the app, and viewing the measured volume appeared to make diary completion more engaging for children. This suggests that a connected measuring device may offer added value beyond digital registration alone.

Nevertheless, the more favorable user experience did not translate into a significant difference in diary completion between formats. The dropout rate of 38.8% was close to the anticipated 40% and comparable to previous pediatric digital diary studies (20, 23). Diary completion in children depends not only on the diary format but also on coordination between parents and multiple caregivers, school routines, hobbies, and other out-of-home activities. Our qualitative findings reflected this complexity: smartphone-based registration was convenient when the parent was present but became more difficult when care was shared or activities occurred outside the home. Although the Diary Pod can store measurements internally until later synchronization with the app, this functionality must be communicated clearly and repeatedly to ensure that families can make effective use of it.

Previous research on digital bladder diaries in pediatric care has identified similar barriers, including coordination with schools and other caregivers, and co-parenting, as well as software maintenance and privacy and security requirements (23). These privacy and security requirements are particularly important because trust in digital health platforms varies across sociodemographic groups (40). Beyond ensuring trustworthy technology, equitable implementation also requires attention to families’ ability to access and use these technologies. Unequal access to suitable devices and affordable connectivity, together with differences in digital literacy, may limit participation and widen existing disparities (41). Addressing these barriers will require multifaceted strategies, with our willingness-to-pay findings indicating that reimbursement could be one important component.

Despite these implementation challenges, the automated diary was clearly preferred by both parents and children in this study and resulted in greater child involvement. While no significant difference in overall diary completion was observed, greater involvement and motivation may still support more consistent and complete registration within individual diaries, potentially improving the reliability of the collected data. Beyond individual diary periods, these advantages may be particularly valuable for sustaining engagement when diary monitoring is repeated as part of a longer-term treatment trajectory. However, these potential effects were not directly assessed in the present study and require further evaluation.

### 4.5 Longitudinal monitoring and progress feedback

The potential value of automated diaries may extend beyond a single diagnostic assessment. Longitudinal progression curves could make it easier for clinicians to compare diaries over time. This may be particularly valuable in difficult or treatment-resistant cases, when evaluating responses to successive therapies, or when monitoring children as they grow. During treatment, symptoms may persist even while relevant diary parameters improve. For example, a child may continue to experience urgency or incontinence while average voided volume increases (Figure 7). Structured longitudinal data could help clinicians recognize these changes, evaluate treatment response, and adjust therapy accordingly.

**Figure 7:**
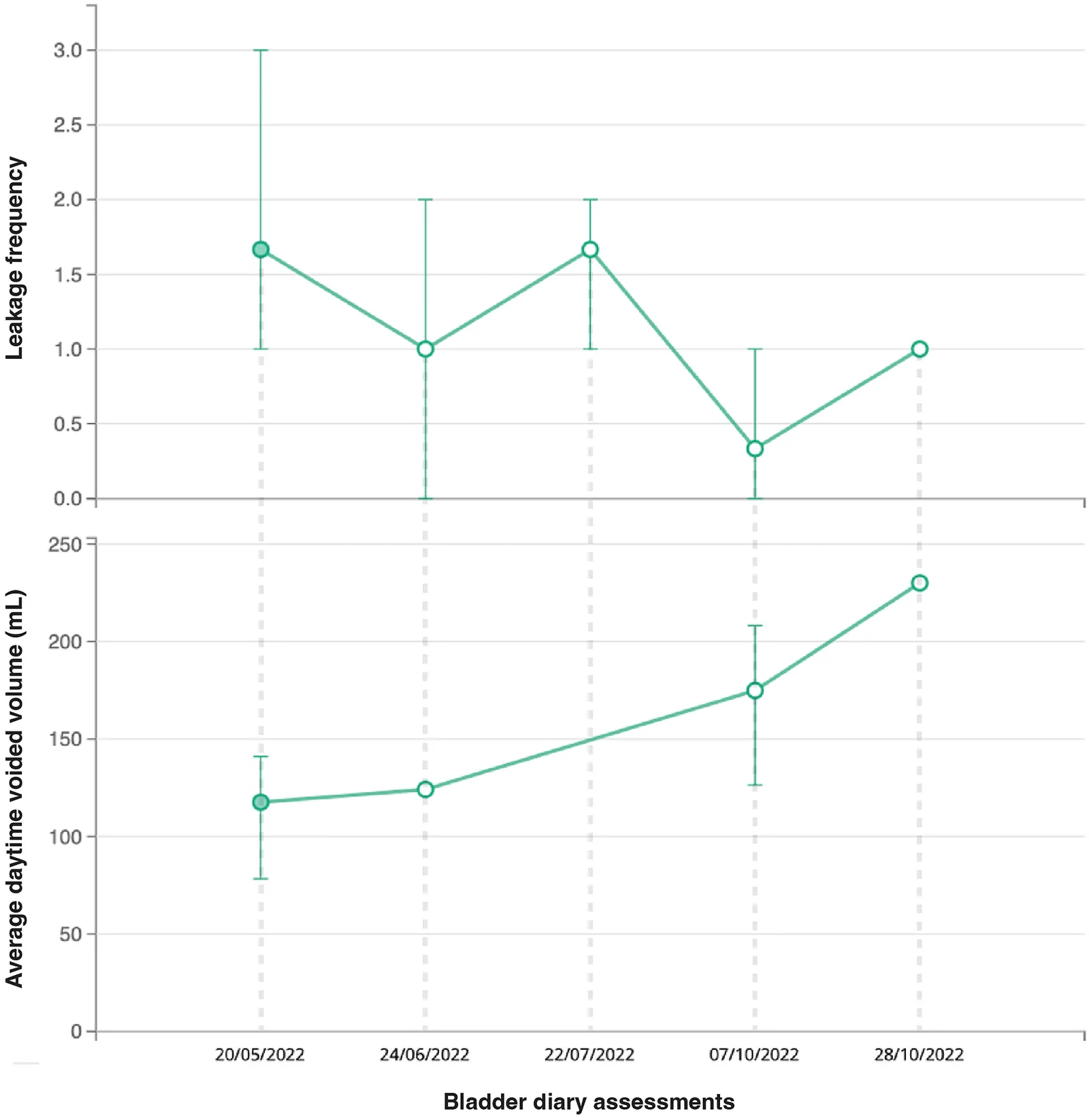
Progress across multiple bladder diaries in an anonymized patient case, showing persistent but decreasing leakage alongside increasing average daytime voided volume.

Making such progress visible may also have motivational value for children and parents. Continued wetting may make it difficult for families to recognize gradual improvement, whereas progression curves could demonstrate changes in underlying diary parameters before complete symptom resolution is achieved. This feedback could provide a more nuanced understanding of treatment progress, support shared discussions about treatment goals, and encourage continued engagement and adherence to treatment.

Parent interviews supported the relevance of this longer-term perspective: the Diary Pod was perceived as more valuable when considered part of a longer-term therapy or training trajectory rather than as a short-term assessment tool. Although conducted in adult men, the Lutastic+ pilot study illustrates the feasibility of integrating the Diary Pod and app into a longer-term digital therapy program, with high engagement and improved LUTS outcomes over 12 weeks (42).

### 4.6 Clinical implementation and recommendations

Our findings suggest that automated bladder diaries may offer the greatest potential added value when diaries must be completed repeatedly. Relevant contexts include complex or treatment-resistant cases, comparisons between therapies, and longer-term digital therapy. In these situations, the ability to compare structured measurements over time and the greater engagement associated with the automated format may help justify the additional cost of the device. For a one-time assessment, a digital diary without a connected measuring device or a conventional paper diary may be sufficient. Regardless of format, clinicians should consider the context and representativeness of the recorded days and provide guidance on suitable measuring jugs with appropriate accuracy and scale resolution given the observed variability in self-provided measuring jugs.

### 4.7 Strengths and limitations

This study has several strengths. The randomized crossover design allowed participants to use both diary formats, reducing between-participant variability and enabling direct comparison of automated and paper diaries in the same child. The random order of diary formats reduced the risk of systematic order effects. The mixed-methods design provided both quantitative data and qualitative insight into the reasons behind observed differences, preferences, and user experiences from the perspectives of clinicians, parents, and children. To our knowledge, this is the first pediatric evaluation of an automated bladder diary.

Several limitations should be acknowledged. First, there was no true reference standard for bladder diary outcomes in the home setting. Therefore, disagreement between formats cannot be definitively attributed to either the automated or the paper diary. Moreover, because the two methods differed in both their measurement and registration procedures, the separate contribution of each component to the observed differences could not be isolated. Rather than standardizing the measuring jugs used with paper diaries, the study evaluated the two complete diary methods as implemented in routine practice at the participating centers. This pragmatic approach captured real-world variation in manual measuring equipment and made the comparison more representative of everyday clinical practice.

Second, the relatively short minimum two-day interval between diary periods was chosen based on clinician feedback to facilitate recruitment and completion within suitable periods, such as school holidays. As no treatment was administered, a formal treatment washout was not considered necessary. However, the interval may have been insufficient for self-monitoring effects to disappear. Although no consistent period or sequence effects emerged at the group level, our qualitative findings suggest that self-monitoring may nevertheless have affected individual participants.

Third, dropout was substantial, although anticipated and comparable to previous pediatric bladder diary studies. This may have introduced selection bias toward more motivated, organized, or technologically confident families who were able to complete both diary formats. Although completers and non-completers were descriptively similar in age, sex, and randomized sequence, information on motivation, smartphone use, and technology familiarity was unavailable for most non-completers and could therefore not be meaningfully compared between groups. Consequently, the usability and preference findings may overrepresent families who were able and willing to engage with both diary formats. This could result in overly favorable findings for the automated diary if less technologically confident families were less likely to complete the study. Conversely, the added value of the automated diary may be underestimated, as families who struggle most with conventional paper diaries may have been less likely to participate or complete the study. However, the direction and magnitude of this potential selection bias cannot be determined from the available data.

Fourth, the usability survey was developed specifically for this study with expert input and internal testing, but it was neither based on a validated instrument nor formally pilot-tested or validated in the target population. This may limit comparability with other studies, although it enabled targeted assessment of usability using complementary quantitative and qualitative data. Furthermore, conclusions regarding child engagement and experience were primarily based on parental observations.

Fifth, data extraction and analyses were performed by the first author, an employee of the device manufacturer, without separate blinded review or independent replication. The involvement of developer-affiliated researchers provided device-specific technical and implementation expertise relevant to this early-stage evaluation of a novel automated diary, but may also have introduced bias. Two academic investigators without financial ties to Minze Health supervised the study and contributed to its design, methodology, and interpretation. Furthermore, clinical diary interpretation was independent of the company: all 49 interpretations were performed by clinicians without financial ties to Minze Health. Nevertheless, bias in data handling and interpretation cannot be excluded. Future independent studies, including real-world evaluations of measurement performance, would complement these findings and further strengthen the evidence base for automated bladder diaries in pediatric practice.

Finally, the study evaluated short-term diary completion only. The proposed benefits for longitudinal monitoring, progress visualization, and sustained engagement therefore remain to be established.

### 4.8 Conclusion

Our findings highlight several potential advantages of the automated diary, including standardized measurement and registration, automatically generated guideline-based calculations, greater child involvement, strong usability, and strong family preference. Together, these features may offer a more engaging and standardized approach to bladder diary completion, particularly for repeated monitoring in complex or treatment-resistant cases and during longer-term digital therapy.

However, individual-level measurement equivalence with the paper diary was not demonstrated, as the limits of agreement exceeded the prespecified margin. In the absence of a true reference standard, the observed disagreement does not establish which format was more accurate. The paper diary served as a conventional comparator rather than a reference standard, and its completion burden, together with the observed variability in self-provided measuring jugs, may affect data completeness and reliability.

Both formats received high clinical usefulness ratings, although clinician preferences were mixed across patients, and paper diaries remained valued for their familiarity and rapid visual overview. Our findings suggest that guideline-based calculations were not consistently used in routine clinical practice, whether they required manual calculation or were generated automatically. Realizing the clinical value of automated bladder diaries will therefore require both effective workflow integration and systematic use of the generated guideline-based outputs by clinicians.

Further independent real-world evaluation is needed to assess the measurement performance of the automated bladder diary and determine whether its potential advantages translate into measurable clinical benefits.

## Data Availability

The datasets presented in this article are not readily available because they contain sensitive health data from children and qualitative data that could permit participant re-identification, and public data sharing was not covered by the informed consent obtained. De-identified quantitative data may be made available by the corresponding author upon reasonable request, subject to applicable ethical and legal requirements. Requests to access the datasets should be directed to lola.bladt@uantwerpen.be.

## References

[1] Von Gontard A Heron J Joinson C. Family history of nocturnal enuresis and urinary incontinence: results from a large epidemiological study. J Urol. (2011) 185:2303–7. 10.1016/j.juro.2011.02.040 21511300

[2] Yeung CK Sreedhar B Sihoe JDY Sit FKY Lau J. Differences in characteristics of nocturnal enuresis between children and adolescents: a critical appraisal from a large epidemiological study. BJU Int. (2006) 97:1069–73. 10.1111/j.1464-410X.2006.06074.x 16643494

[3] Linde JM Nijman RJM Trzpis M Broens PMA. Prevalence of urinary incontinence and other lower urinary tract symptoms in children in the Netherlands. J Pediatr Urol. (2019) 15:164.e1–e7. 10.1016/j.jpurol.2018.10.027 30583907

[4] Shrestha N Sahukhala S K CD Sandalcidi D Adhikari SP. Prevalence of urinary incontinence in school going children: a cross-sectional study. J Nepal Health Res Counc. (2021) 18:676–80. 10.33314/jnhrc.v18i4.2506 33510509

[5] Austin PF Bauer SB Bower W Chase J Franco I Hoebeke P. The standardization of terminology of lower urinary tract function in children and adolescents: update report from the Standardization Committee of the International Children’s Continence Society. Neurourol Urodyn. (2016) 35:471–81. 10.1002/nau.22751 25772695

[6] Nevéus T Fonseca E Franco I Kawauchi A Kovacevic L Nieuwhof-Leppink A. Management and treatment of nocturnal enuresis—an updated standardization document from the International Children’s Continence Society. J Pediatr Urol. (2020) 16:10–9. 10.1016/j.jpurol.2019.12.020 32278657

[7] Hoebeke P Bower W Combs A De Jong T Yang S. Diagnostic evaluation of children with daytime incontinence. J Urol. (2010) 183:699–703. 10.1016/j.juro.2009.10.038 20022025

[8] Chang S-J Van Laecke E Bauer SB Von Gontard A Bagli D Bower WF. Treatment of daytime urinary incontinence: a standardization document from the International Children’s Continence Society. Neurourol Urodyn. (2017) 36:43–50. 10.1002/nau.22911 26473630

[9] Jørgensen CS Dossche L Zhai R Maternik M Kamperis K Breinbjerg AS. Development of a novel prediction tool for response to first-line treatments of monosymptomatic nocturnal enuresis: a randomized, controlled, international, multicenter study (DRYCHILD). J Urol. (2024) 212:539–49. 10.1097/JU.0000000000004129 38950376

[10] Flynn KE Wiseman JB Helmuth ME Smith AR Bradley CS Cameron AP. Comparing clinical bladder diaries and recalled patient reports for measuring lower urinary tract symptoms in the Symptoms of Lower Urinary Tract Dysfunction Research Network (LURN). Neurourol Urodyn. (2022) 41:1711–21. 10.1002/nau.25030 36066068 PMC9633398

[11] Tincello DG Williams KS Joshi M Assassa RP Abrams KR. Urinary diaries: a comparison of data collected for three days versus seven days. Obstet Gynecol. (2007) 109:277–80. 10.1097/01.AOG.0000252832.21986.c8 17267824

[12] Haylen BT De Ridder D Freeman RM Swift SE Berghmans B Lee J. An International Urogynecological Association (IUGA)/International Continence Society (ICS) joint report on the terminology for female pelvic floor dysfunction. Int Urogynecol J. (2010) 21:5–26. 10.1007/s00192-009-0976-9 19937315

[13] Hashim H Blanker MH Drake MJ Djurhuus JC Meijlink J Morris V. International Continence Society (ICS) report on the terminology for nocturia and nocturnal lower urinary tract function. Neurourol Urodyn. (2019) 38:499–508. 10.1002/nau.23917 30644584

[14] Mateu Arrom L Peri Cusi L Franco de Castro A López-Fando L Jiménez Cidre MÁ Alcaraz Asensio A. Development and feasibility assessment of a 3 day electronic bladder diary as an app for smart-phone. Neurourol Urodyn. (2018) 37:1717–23. 10.1002/nau.23494 29356063

[15] Vaccari NA Da Silveira LTY Bortolini MAT Haddad JM Baracat EC Ferreira EAG. Content and functionality features of voiding diary applications for mobile devices in Brazil: a descriptive analysis. Int Urogynecol J. (2020) 31:2573–81. 10.1007/s00192-020-04382-6 32592019

[16] Myint M Adam A Herath S Smith G. Mobile phone applications in management of enuresis: the good, the bad, and the unreliable!. J Pediatr Urol. (2016) 12:112.e1–e6. 10.1016/j.jpurol.2015.09.011 26611873

[17] Rabin JM McNett J Badlani GH. “Compu-void II”: the computerized voiding diary. J Med Syst. (1996) 20:19–34. 10.1007/BF02260871 8708489

[18] Quinn P Goka J Richardson H. Assessment of an electronic daily diary in patients with overactive bladder. BJU Int. (2003) 91:647–52. 10.1046/j.1464-410X.2003.04168.x 12699477

[19] Mangera A Marzo A Heron N Fernando D Hameed K Soliman A-HA. Development of two electronic bladder diaries: a patient and healthcare professionals pilot study. Neurourol Urodyn. (2014) 33:1101–9. 10.1002/nau.22469 24000163

[20] Johnson EK Estrada CR Johnson KL Nguyen HT Rosoklija I Nelson CP. Evaluation of a mobile voiding diary for pediatric patients with voiding dysfunction: a prospective comparative study. J Urol. (2014) 192:908–13. 10.1016/j.juro.2014.03.099 24704008 PMC4143466

[21] Abrams P Paty J Martina R Newgreen DT Van Maanen R Paireddy A. Electronic bladder diaries of differing duration versus a paper diary for data collection in overactive bladder. Neurourol Urodyn. (2016) 35:743–9. 10.1002/nau.22800 26174907

[22] Sussman RD Richter LA Tefera E Park AJ Sokol AI Gutman RE. Utilizing technology in assessment of lower urinary tract symptoms: a randomized trial of electronic versus paper voiding diaries. Female Pelvic Med Reconstr Surg. (2016) 22:224–8. 10.1097/SPV.0000000000000266 27054797

[23] De Wall LL Kragt EA Van De Wetering EH Cobussen-Boekhorst JG Mantel-van Stel J Kortmann BB. Challenges in e-health: the effect of digitalisation of frequency voiding charts on compliance in paediatric patients. Randomised controlled trial comparing digital and versus paper frequency voiding charts. Digit Health. (2023) 9:20552076231207577. 10.1177/20552076231207577 37954691 PMC10634267

[24] Liapis I Toozs-Hobson P. Preliminary results of a clinical trial using a novel device for the automation of measurement of voided volume in the bladder diaries of women with lower urinary tract symptoms (e-BlaDR study). In: IUGA 48th Annual Meeting (The Hague, the Netherlands) (2023).

[25] International Organisation for Standardization. ISO 7056: 1981 Plastics laboratory ware – beakers. BSI Standards Publication (1981).

[26] International Organisation for Standardization. BS ISO 3819:1985 Laboratory glassware – beakers. BSI Standards Publication (1985).

[27] Li M Tsai Y-C Yang SS-D Pong Y-H Tsai Y-T Tsai VF-S. The evolution of uroflowmetry and bladder diary and the emerging trend of using home devices from hospital to home. Interact J Med Res. (2025) 14:e66694. 10.2196/66694 39874564 PMC11815292

[28] Williamson J Qayyum T Bryan N Blunt L. UScale: a digital device for automatic urine volume measurement and frequency volume charting. Ther Adv Urol. (2019) 11:1756287219875586. 10.1177/1756287219875586 31565071 PMC6755625

[29] Okumura Y Amaya Y Inamura S Inamura M Kaeriyama K Okubo N. Usability and accuracy of a novel automatic bladder diary device (Uro-diary checker). Urology. (2026) 214:20–4. 10.1016/j.urology.2026.06.011 42264191

[30] Bladt L De Win G De Wachter S. Accuracy validation of a novel automated bladder diary device, Minze Diary Pod. Eur Urol. (2022) 81:S151. 10.1016/S0302-2838(22)00189-0

[31] Perrouin-Verbe M Charrier A De Guerry C Simon A Puisset S Queval L. Contribution of the DIARY POD during the test phase of sacral nerve modulation in refractory overactive bladder: a bicentric study. In: 47th Annual Congress of the SIFUD-PP (Biarritz, France) (2025).

[32] Alameda F Audenet F Mandron E Timsit MO Fontaine E Pietak M. Étude CamiCon: évaluation d’un nouvel outil pour la réalisation d’un calendrier mictionnel automatisé et connecté. French J Urol. (2024) 34:102582. 10.1016/j.fjurol.2024.102582 38364362

[33] Liapis I Toozs-Hobson P. Does automation of voided volume calculation improve the accuracy of bladder diaries? An update on the E-bladr study results. In: IUGA 49th Annual Meeting (Singapore) (2024).

[34] Schulte-Baukloh H Weiss C Höppner D Haberecht K Borgmann H Ralla B. Real-life monitoring: precise onset of action after BotulinumtoxinA injection. JU Open Plus. (2025) 3. 10.1097/JU9.0000000000000277

[35] Mateu Arrom L Peri Cusi L López-Fando L Franco de Castro A Jiménez Cidre MÁ Alcaraz A. Validation of a 3 day electronic bladder diary as an app for smart-phone. Neurourol Urodyn. (2019) 38:764–9. 10.1002/nau.23914 30620092

[36] Jørgensen CS Pedersen SN Dossche L Zhai R Maternik M Raes A. Intra-individual variability in voiding diaries of children with enuresis. J Pediatr Urol. (2025) 21:356–61. 10.1016/j.jpurol.2024.11.020 39675941

[37] Bryan NP Chapple CR. Frequency volume charts in the assessment and evaluation of treatment: how should we use them? Eur Urol. (2004) 46:636–40. 10.1016/j.eururo.2004.07.023 15474275

[38] Kaga K Mikami K Kaga M. Utilizing bladder diaries to prevent unnecessary treatment in patients with storage dysfunction: a retrospective study. Cureus. (2024) 16:e68437. 10.7759/cureus.68437 39360064 PMC11445800

[39] Nieuwhof-Leppink AJ Hussong J Chase J Larsson J Renson C Hoebeke P. Definitions, indications and practice of urotherapy in children and adolescents: - a standardization document of the International Children’s Continence Society (ICCS). J Pediatr Urol. (2021) 17:172–81. 10.1016/j.jpurol.2020.11.006 33478902

[40] Chow E Virani A Pinkney S Abdulhussein FS Van Rooij T Görges M. Caregiver and youth characteristics that influence trust in digital health platforms in pediatric care: mixed methods study. J Med Internet Res. (2024) 26:e53657. 10.2196/53657 39467279 PMC11555442

[41] Whitehead L Robinson S Arabiat D Jenkins M Morelius E. The report of access and engagement with digital health interventions among children and young people: systematic review. JMIR Pediatr Parent. (2024) 7:e44199. 10.2196/44199 38231560 PMC10831666

[42] Bladt L Vermeulen Z Vermeulen J Van Campenhout L Vermandel A De Wachter S. High adherence and improved male lower urinary tract symptoms: a pilot study of the digital therapeutic Lutastic+. Int J Reconstr Urol. (2026) 4:131–9. 10.4103/IJRU.IJRU_8_26

