# Supplementary material for: Comparison of an automated and paper bladder diary in pediatric patients: mixed-methods, randomized crossover study
Source: Front Pediatr. 2026 Oct 8;14:1959698. doi: 10.3389/fped.2026.1959698 (PMC13687010; doi:10.3389/fped.2026.1959698)
Supplement: Supplementary file 1 [file Supplementaryfile1.docx]

Supplementary Figures and Tables

**Supplementary Figure 1.** Example study package for the automated-first sequence, showing the Diary Pod, written instructions, and locked document pouch used to conceal the second diary format until completion of the first diary period.

**Supplementary Table S1.** Linear mixed-effects crossover analyses of diary outcomes

| **Outcome** | **Participants (observations)** | **Method:**  **Automated - Paper, estimate (95% CI); p** | **Period:**  **Period 2 - Period 1, estimate (95% CI); p** | **Sequence: Automated first – Paper first, estimate (95% CI); p** |
| --- | --- | --- | --- | --- |
| Average daytime voided volume, mL | 49 (97) | -2.7 (-10.8 to 5.4); 0.508 | 5.1 (-3.1 to 13.2);  0.215 | -15.2 (-49.8 to 19.4); 0.382 |
| Daytime frequency | 49 (98) | 0.01 (-0.4 to 0.4);  0.952 | -0.3 (-0.7 to 0.1);  0.148 | -0.4 (-1.2 to 0.5);  0.392 |
| Nighttime frequency | 49 (98) | 0.2 (-0.04 to 0.3);  0.112 | 0.2 (-0.02 to 0.4); 0.075 | -0.1 (-0.4 to 0.1);  0.344 |
| Urgency frequency | 49 (98) | -1.1 (-1.7 to -0.6); 0.0002 | -0.2 (-0.7 to 0.4);  0.563 | -0.1 (-0.9 to 0.7);  0.776 |
| Leakage frequency | 49 (98) | -0.1 (-0.4 to 0.2);  0.353 | -0.2 (-0.5 to 0.1);  0.195 | 0.3 (-0.3 to 0.8);  0.303 |

Estimates were obtained from linear mixed-effects models including diary method, period, and randomized sequence as fixed effects and participant as a random intercept. Positive estimates indicate higher values for automated versus paper, period 2 versus period 1, and the Automated first versus Paper first sequence, respectively. CI, confidence interval.

**Supplementary Table S2.** Paired parent-reported experience with the automated and paper bladder diaries.

| **Item** | **Automated**  **(N = 47), n (%)** | **Paper**  **(N = 47), n (%)** | **Absolute difference, percentage points** | **Discordant pairs, automated only : paper only** | **p-valueᵃ** |
| --- | --- | --- | --- | --- | --- |
| Motivation | 41 (87.2) | 34 (72.3) | +14.9 | 8 : 1 | **0.0391** |
| Pleasant use | 34 (72.3) | 12 (25.5) | +46.8 | 22 : 0 | **4.77x10^-7^** |
| Child involvement | 47 (100.0) | 36 (76.6) | +23.4 | 11 : 0 | **0.000977** |
| Comfortable voiding | 32 (68.1) | 30 (63.8) | +4.3 | 11 : 9 | 0.824 |
| Hygienic use | 31 (66.0) | 30 (63.8) | +2.1 | 9 : 8 | 1.000 |
| Events easy to record | 42 (89.4) | 37 (78.7) | +10.6 | 7 : 2 | 0.180 |
| Events consistently recorded | 35 (74.5) | 33 (70.2) | +4.3 | 6 : 4 | 0.754 |
| Perceived error-free use | 25 (53.2) | 13 (27.7) | +25.5 | 16 : 4 | **0.0118** |
| Discretion | 24 (51.1) | 14 (29.8) | +21.3 | 16 : 6 | 0.0525 |

ᵃ P values were calculated using two-sided McNemar tests based on discordant pairs. Bold values indicate statistical significance at p < 0.05.

Percentages are based on 47 participants with responses for both diary formats.

Positive responses were defined as scores of 4–5 on a 5-point Likert scale after reverse-coding negatively phrased items.
